# Mediterranean diet adherence and systemic inflammation in people with HIV and PrEP users

**DOI:** 10.3389/fnut.2026.1860061

**Published:** 2026-07-16

**Authors:** Mónica Manzano, Yolanda Campanero, Sergio Sabogal, Ana M. Lorenzo-Mora, Alicia Simón-Rueda, Montserrat Torres, Adrián Valls-Carbó, Mayte Coiras, Vicente Estrada

**Affiliations:** 1 Department of Pharmacy and Nutrition, Faculty of Biomedical and Health Sciences, Universidad Europea de Madrid, Madrid, Spain; 2 Infectious Diseases Unit, Hospital Clínico San Carlos, IdISSC, Madrid, Spain; 3 Immunopathology and Viral Reservoir Unit, National Center of Microbiology, Instituto de Salud Carlos III, Majadahonda, Madrid, Spain; 4 Research in Medical-Surgical Sciences, Universidad Complutense de Madrid (UCM), Madrid, Spain; 5 Biomedical Research Center Network in Infectious Diseases (CIBERINFEC), Instituto de Salud Carlos III, Madrid, Spain; 6 Biomedical Sciences and Public Health, Universidad Nacional de Educación a Distancia (UNED), Madrid, Spain; 7 Faculty of Medicine, Universidad Complutense de Madrid, Madrid, Spain

**Keywords:** Mediterranean diet, cytokines, inflammation, HIV, PrEP, MED-DQI

## Abstract

**Background:**

Chronic low-grade inflammation contributes to long-term morbidity in people with HIV (PWH). Individuals using pre-exposure prophylaxis (PrEP) may share lifestyle factors associated with immune activation. We evaluated whether adherence to the Mediterranean diet, assessed using the Mediterranean Diet Quality Index (MED-DQI), is associated with inflammation in PWH and PrEP users.

**Methods:**

We conducted a cross-sectional study of 82 MSM (24 PWH and 58 PrEP users). We calculated MED-DQI from 3-day dietary records and measured 45 plasma cytokines by multiplex immunoassay. Cytokine values were ln-transformed, standardized to *Z*-scores, and combined into a global inflammatory index as the primary outcome. We analyzed associations using Student’s *t*-test, odds ratios (OR), and multivariable linear regression, adjusting for age, BMI, physical activity, alcohol intake, total energy intake, and recreational drug use.

**Results:**

Diet quality did not differ between groups (PWH 7.3 ± 1.9; PrEP 7.4 ± 1.6). Participants with lower adherence (MED-DQI ≥ 8) had a higher inflammatory index than those with better adherence (MED-DQI ≤ 7) (mean difference −0.34; 95% CI −0.62 to −0.07; *p* = 0.016) and higher odds of elevated inflammation (OR 2.46; 95% CI 1.01 to 6.02; *p* = 0.038). Of the 45 cytokines analyzed, 24 were significantly associated with MED-DQI (*p* < 0.05), and all remained significant after FDR correction (Benjamini–Hochberg; qFDR < 0.10). Multiple linear regression analyses confirmed these findings; IL-17A was associated with an approximately 18% higher concentration per one-point increase in MED-DQI (*β* = 0.165), reflecting greater inflammation with poorer adherence to the Mediterranean diet.

**Discussion:**

Our study found that adherence to the Mediterranean diet was associated with a lower global inflammatory index in both PWH and PrEP users. Lower adherence was associated with higher global inflammatory index values and elevated cytokine concentrations, suggesting differences in systemic immune activation. Even modest differences in diet quality were reflected in variations across the cytokine profile. No differences were observed between groups, suggesting these patterns were more closely associated with diet quality than group status. Diet quality may be a modifiable factor associated with inflammatory profiles. Mediterranean dietary approaches could represent a strategy to address chronic low-grade inflammation.

## 1 Introduction

The Mediterranean diet is widely recognized for its anti-inflammatory and immunomodulatory properties; however, its influence on comprehensive cytokine profiles in people living with HIV (PWH) or individuals at risk of acquiring HIV remains limited (1–4).

Chronic inflammation plays a key role in the pathophysiology of major inflammatory and metabolic diseases, generating increasing interest in understanding how dietary patterns modulate immune and inflammatory biomarkers. In PWH, persistent immune activation and immunosenescence contribute to excess morbidity and mortality (5–7). Similarly, individuals using pre-exposure prophylaxis (PrEP), although HIV-negative, share with PWH certain behavioral and lifestyle characteristics, including sexual practices, psychosocial stress and patterns of recreational substance use, which may influence immune responses (8–10). In this context, the Mediterranean diet, rich in antioxidants, monounsaturated fatty acids, and polyphenols, has demonstrated benefits in cardiometabolic and neurodegenerative conditions and may modulate systemic inflammatory pathways (11–16). Previous research identified associations between dietary patterns, gut microbiota composition, and selected inflammatory biomarkers in an independent cohort of PWH. However, that study did not include PrEP users or evaluate broader cytokine-derived inflammatory profiles in relation to Mediterranean diet adherence (17). Studies examining its association with detailed inflammatory cytokine patterns in PWH and PrEP users remain limited.

## 2 Methods

### 2.1 Study design and sample collection

We conducted a cross-sectional study and recruited participants between February 2023 and February 2024 at the Infectious Diseases and HIV Unit of Hospital Clínico San Carlos in Madrid, Spain. The study included two groups: PWH and PrEP. All participants were aged ≥18 years.

For the PWH group, inclusion criteria were confirmed HIV infection and receipt of antiretroviral therapy (ART) for at least 1 year with an undetectable viral load at enrollment. We excluded individuals with acute infection at recruitment, antibiotic use within the previous month, or conditions that could compromise safety, interfere with study assessments, or affect adherence to study procedures.

All participants provided written informed consent. The study protocol was approved by the Ethics Committee of Hospital Clínico San Carlos (Ref. C.I. 24/020-E) and conducted in accordance with the Declaration of Helsinki (2013) and Good Clinical Practice guidelines. We collected dietary intake data, physical activity information, and blood samples. Participants completed 3-day dietary records using the REDCap (Research Electronic Data Capture) electronic system hosted at Hospital Clínico San Carlos. Body Mass Index (BMI) was measured for all participants.

### 2.2 Dietary data

A three-day dietary record (18), including two weekdays and one weekend day, was used to collect information on all foods, beverages, and food supplements consumed by participants. To ensure accurate reporting, participants received written instructions detailing how to record foods, ingredients, cooking methods, brand names, and portion sizes using household measures. Nutritionists from the research team reviewed all dietary records to minimize errors, such as unrealistic portion sizes, insufficient fluid intake, or inconsistencies in reporting. We processed dietary information using the DIAL software version 3.0.0.12 (Alce Ingeniería, Madrid, Spain), based on data from the Spanish Food Composition Tables (19). This analysis generated detailed tables of total energy intake, macronutrient distribution, and micronutrient intakes (20).

### 2.3 Physical activity data

We collected physical activity data using a questionnaire in which participants recorded the number of hours spent in different activities over a 24-h period, both on working days and weekends, ensuring that the total added up to 24 h.

From these data, we calculated an individual activity coefficient (CAFI), a widely used indicator of overall physical activity level (21, 22). This coefficient served as a global adjustment for physical activity, without detailed characterization of activity types or intensities.

### 2.4 Measurements in blood samples

Peripheral blood samples were obtained from all study participants in EDTA tubes. Plasma was separated by density gradient centrifugation (Ficoll–Hypaque) and stored at −80 °C until analysis. We used 25 μL of plasma to quantify the concentrations of 45 cytokines using a magnetic bead–based multiplex immunoassay on the Luminex xMAP platform (ProcartaPlex™, Thermo Fisher Scientific, Waltham, MA, USA), according to the manufacturer’s instructions.

We classified cytokines according to their predominant immunological role as:

#### 2.4.1 Pro-inflammatory

Interleukin-1 alpha (IL-1α), interleukin-1 beta (IL-1β), interleukin-2 (IL-2), interleukin-7 (IL-7), interleukin-8 (IL-8/CXCL8), interleukin-9 (IL-9), interleukin-12p70 (IL-12p70), interleukin-15 (IL-15), interleukin-17A (IL-17A), interleukin-18 (IL-18), interleukin-23 (IL-23), tumor necrosis factor-alpha (TNF-α), tumor necrosis factor-beta (TNF-β), interferon-gamma (IFN-γ), interferon-alpha (IFN-α), interferon-gamma–induced protein 10 (IP-10/CXCL10), eotaxin (CCL11), growth-related oncogene-alpha (GRO-α/CXCL1), monocyte chemoattractant protein-1 (MCP-1/CCL2), macrophage inflammatory protein-1 alpha (MIP-1α/CCL3), and macrophage inflammatory protein-1 beta (MIP-1β/CCL4).

#### 2.4.2 Anti-inflammatory

Interleukin-4 (IL-4), interleukin-5 (IL-5), interleukin-10 (IL-10), interleukin-13 (IL-13), interleukin-27 (IL-27), interleukin-1 receptor antagonist (IL-1RA), and leukemia inhibitory factor (LIF).

#### 2.4.3 Mixed or regulatory

Interleukin-6 (IL-6), interleukin-21 (IL-21), interleukin-22 (IL-22), interleukin-31 (IL-31), regulated on activation, normal T cell expressed and secreted (RANTES/CCL5), nerve growth factor-beta (NGF-β), stem cell factor (SCF), granulocyte–macrophage colony-stimulating factor (GM-CSF), stromal cell-derived factor-1 alpha (SDF-1α/CXCL12), platelet-derived growth factor-BB (PDGF-BB), vascular endothelial growth factor-A (VEGF-A), vascular endothelial growth factor-D (VEGF-D), fibroblast growth factor-2 (FGF-2), hepatocyte growth factor (HGF), brain-derived neurotrophic factor (BDNF), epidermal growth factor (EGF) and, placental growth factor-1 (PlGF-1).

Cytokines were grouped based on their predominant functional roles described in chronic systemic inflammation, nutritional immunology, and HIV-related immune dysregulation. We acknowledge that several cytokines exert context-dependent effects; therefore, this classification reflects dominant functional patterns rather than absolute categories. This approach is consistent with prior studies evaluating global inflammatory burden and diet-related immunomodulation (23).

### 2.5 Mediterranean Diet Quality Index (MED-DQI)

To assess diet quality, we used the MED-DQI, which is considered one of the most appropriate indexes for evaluating adherence to the Mediterranean dietary pattern in adults (24–26). The index includes the following components: percentage of total energy derived from saturated fatty acids, cholesterol (mg), meat (g), olive oil (mL), fish (g), and fruits and vegetables (g). Each component is scored 0, 1, or 2 according to established recommendations. MED-DQI scores between 1 and 4 are considered good (G1), 5–7 moderately good (G2), 8–10 moderately poor (G3), and 11–14 poor (G4). These cutoffs are consistent with the scoring system originally described for the MED-DQI and have been applied in adult populations in previous studies (25).

### 2.6 Statistical analysis

We presented results as mean ± standard deviation (SD) or median (25th–75th percentile) for continuous variables, and as absolute frequencies and percentages for categorical variables. We transformed cytokine concentrations using the natural logarithm (ln) to reduce skewness and stabilize variance, and then standardized these values as *Z*-scores.

We derived a global inflammatory index as the arithmetic mean of the *Z*-scores calculated from the 45 ln-transformed cytokine concentrations. This index includes pro-inflammatory, anti-inflammatory, and mixed/regulatory cytokines and provides a summary measure of the overall cytokine profile, which may reflect differences in systemic immune activation across participants. We compared this index between PWH and PrEP users using Student’s *t*-test.

We calculated mean cytokine *Z*-scores according to functional classification (pro-inflammatory, anti-inflammatory, and mixed/pleiotropic). For group comparisons, we grouped participants into higher (G1–G2) and lower (G3–G4) adherence categories and used Welch’s *t*-test.

To explore associations, we analyzed the relationship between MED-DQI and individual cytokines using two-tailed Pearson correlations. We considered a nominal *p* value <0.05 statistically significant. To address multiple comparisons across the 45 cytokines analyzed, we applied the Benjamini–Hochberg false discovery rate (FDR) correction and considered FDR-adjusted *p*-values (*q*-values) < 0.10 statistically significant for both Pearson correlations and multiple linear regression analyses, given the exploratory nature of the study, the modest sample size, and the large number of biomarkers evaluated.

To estimate the odds of a high inflammatory index, defined as values above the median of global inflammatory index, we conducted an odds ratio (OR) analysis comparing participants with lower (MED-DQI ≥ 8) and higher (MED-DQI ≤ 7) adherence to the Mediterranean diet.

To assess the association between MED-DQI, as an indicator of Mediterranean diet adherence, and inflammatory biomarker concentrations, we used multiple linear regression models. *Z*-scores of ln-transformed cytokine concentrations served as dependent variables, with the MED-DQI score as the main predictor. MED-DQI was analyzed on its original scale, where each additional point represents poorer adherence to the Mediterranean diet. We adjusted all models for age, BMI, physical activity level (CAFI), alcohol intake, total energy intake, and recreational drug use. To evaluate confounding, we applied the change-in-estimate criterion (*Δβ*), defined as the percentage change in the exposure coefficient after covariate adjustment, and prespecified *Δβ* < 10% as negligible and 10–20% as indicative of modest confounding.

Finally, we estimated regression coefficients from ln-transformed models and expressed them as the percentage change per one-point increase in MED-DQI (%*Δ*), together with their 95% confidence intervals, using robust (HC3) standard errors. We visualized the results using forest plots, with pro-inflammatory, anti-inflammatory, and mixed/pleiotropic cytokines color-coded as red, green, and blue, respectively.

## 3 Results

### 3.1 Description of the general population

We included 82 male participants, 24 of whom were PWH and 58 were PrEP users, with a median age of 38 years. All participants showed full treatment adherence, and the median BMI was 24.5 kg/m^2^ (Table 1).

**Table 1:** Characteristics of the study population.

Characteristics	PWH (*n* = 24)	PrEP (*n* = 58)	Total (*n* = 82)
Age (median, IQR)	38.9 (33.8–44.0)	38.0 (33.0–44.0)	38.0 (33–43.7)
Male sex (%)	100%	100%	100%
BMI (median; IQR)	24.53 (22.85–26.22)	24.49 (22.89–26.19)	24.50 (22.90–26.18)
Treatment adherence (%)	100%	100%	100%
Years of HIV infection (median; IQR)	10.5 (7–13.3)	–	–
Years in HIV treatment (median; IQR)	9.5 (6.8–13.0)	–	–
CD4 (cells/μL median; IQR)	809 (600–910)	–	–
CD8 (cells/μL median; IQR)	880 (627–1,107)	–	–
CD4/CD8 (median; IQR)	0.9 (0.7–1.0)	–	–
Years on PrEP (median; IQR)	–	3.0 (2.3–4.0)	–

Results are presented as median (25th–75th percentile) when variables are continuous or *n* (%) when categorical. PWH, people living with HIV; PrEP, pre-exposure prophylaxis; BMI, Body Mass Index.

### 3.2 Inflammation and MED-DQI according to study group

To integrate information from the full panel of 45 cytokines, we constructed a composite inflammatory index. After natural-log transformation (ln) of plasma cytokine concentrations, we calculated *Z*-scores to place all biomarkers on a comparable scale. We then derived the Inflammatory Index for each participant as the arithmetic mean of the *Z*-scores of the 45 ln-transformed cytokines. This composite variable provides a summary measure of the overall cytokine profile and may reflect differences in systemic immune activation across individuals.

We compared the Inflammation Index between PWH and PrEP users. Levene’s test indicated homogeneous variances between groups (*F* = 0.017, *p* = 0.897), so the *t*-test assuming equal variances was applied. No significant differences were observed between the two groups [*t*(80) = −0.10, *p* = 0.919]. The mean difference was minimal (−0.016; 95% CI: −0.330 to 0.298), indicating that the overall inflammatory profile did not differ between groups in this sample.

We also assessed diet quality using the Mediterranean Diet Quality Index (MED-DQI), where higher scores indicate poorer adherence to the Mediterranean dietary pattern, and found no significant differences between study groups. PWH showed a mean MED-DQI score of 7.25 ± 1.87, while the PrEP group presented a mean score of 7.33 ± 1.65, indicating comparable adherence to the Mediterranean dietary pattern. The corresponding median (*p*25–*p*75) values were 7 (6–9) for PWH and 7 (6–9) for PrEP, confirming similar diet quality across participants.

We analyzed the distribution of participants by MED-DQI categories in PWH and PrEP groups. Both groups showed predominantly moderate adherence to the Mediterranean dietary pattern. Among PWH, 50.0% were classified as moderately good and 37.5% as moderately poor, whereas in the PrEP group, 51.7 and 44.8% fell within the moderately good and moderately poor categories, respectively. Only a small proportion of participants showed high adherence (good: 8.3% in PWH and 1.7% in PrEP) or poor diet quality (4.2 and 1.7%, respectively) (Table 2).

**Table 2:** Distribution of participants by MED-DQI categories in PWH and PrEP groups.

MED-DQI category	PWH (*n* = 24)	PrEP (*n* = 58)	Total (*n* = 82)
Good (1–4)	2 (8.3%)	1 (1.7%)	3 (3.7%)
Moderately good (5–7)	12 (50.0%)	30 (51.7%)	42 (51.2%)
Moderately poor (8–10)	9 (37.5%)	26 (44.8%)	35 (42.7%)
Poor (11–14)	1 (4.2%)	1 (1.7%)	2 (2.4%)

Data are presented as *n* (%). Percentages may not total 100 due to rounding.

Overall, the PrEP group tended to show slightly lower adherence to the Mediterranean dietary pattern than PWH, although the difference between groups was not statistically significant (*χ*^2^ test, *p* > 0.05).

Given the limited differences in Mediterranean diet adherence between PWH and PrEP users, we performed a comprehensive assessment of inflammatory status based on the degree of adherence to the Mediterranean diet, in order to identify subtle variations in immune activation that might remain undetected in group-based analyses. This approach enhances our understanding of diet–inflammation interactions and provides a basis for developing precision nutrition strategies aimed at modulating chronic inflammation.

### 3.3 Association between MED-DQI and systemic inflammatory cytokines profiles

**Figure 1:**
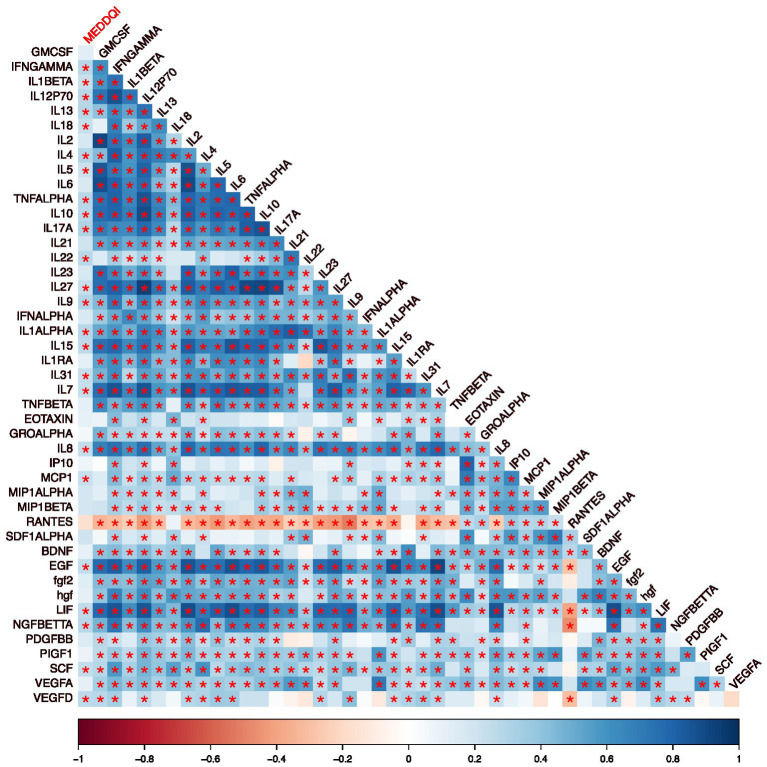
Heatmap of Pearson correlation coefficients between the MED-DQI and plasma inflammatory cytokines. Color intensity reflects the strength and direction of correlations (blue, positive; red, negative).

We observed consistent associations between MED-DQI scores and multiple inflammatory cytokines. In Pearson correlation analyses, 24 cytokines showed significant positive correlations with MED-DQI (*p* < 0.05), indicating that poorer adherence to the Mediterranean diet was associated with higher plasma concentrations of inflammatory cytokines (Supplementary Table 1). After applying the Benjamini–Hochberg false discovery rate (FDR) correction (*m* = 45), all 24 cytokines remained significant (*q* < 0.10), supporting the robustness of this association pattern. Ten cytokines, including IL-17A, TNF-α, IFN-γ, IL-12p70, IL-1α, IL-22, IL-10, IL-9, IL-4, and IL-13, showed qFDR values close to 0.05 (qFDR ≤ 0.053). The strongest correlations were observed for IL-17A (*r* = 0.30, *p* = 0.005), IL-9 (*r* = 0.30, *p* = 0.006), and IL-4 (*r* = 0.29, *p* = 0.008) (Figure 1).

**Figure 2:**
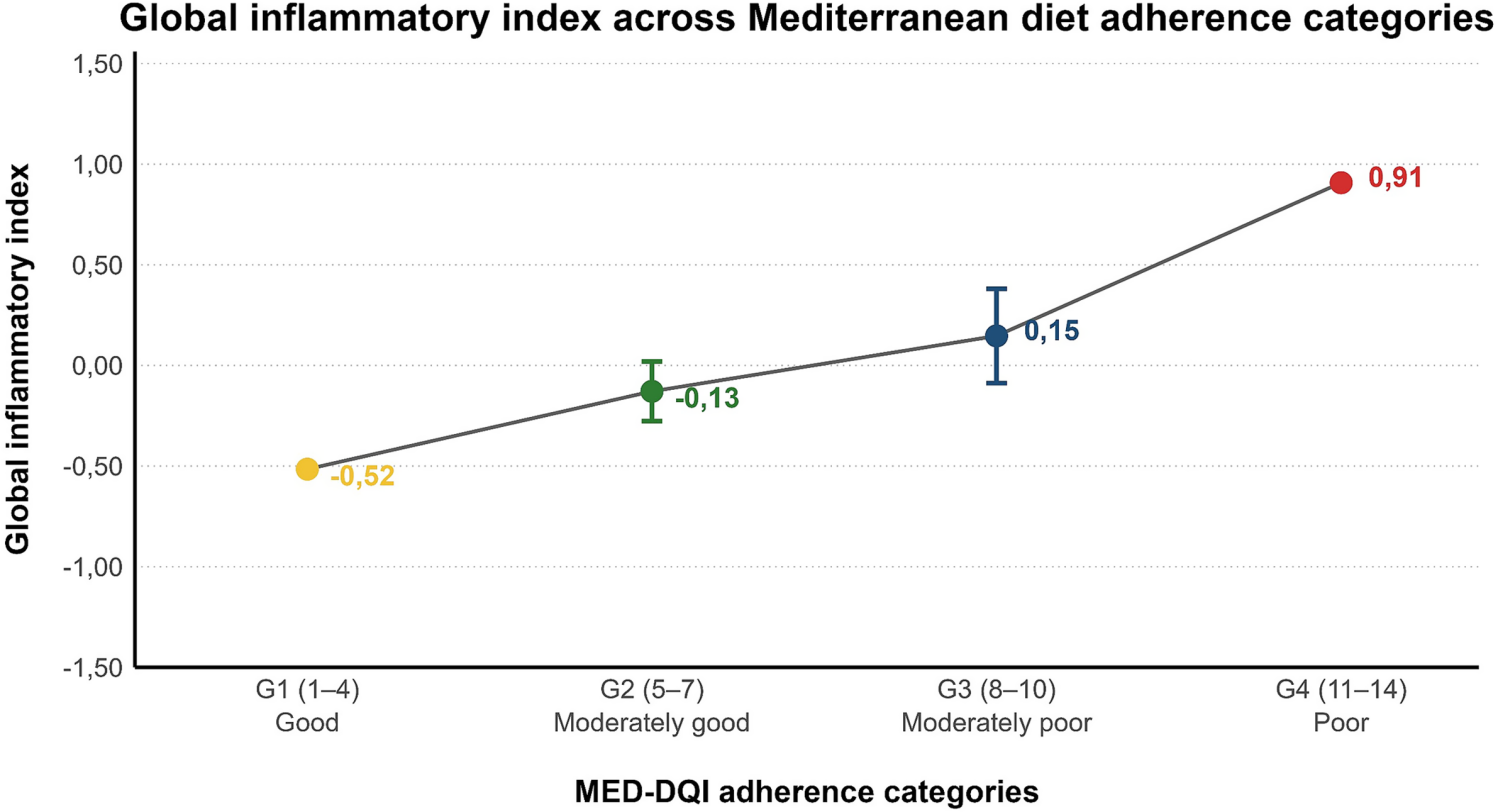
Global inflammatory index across MED-DQI categories. Values are presented as mean ± 95% CI. Inflammatory index increases progressively from “good” to “poor” categories. Group sizes were: good (*n* = 3), moderately good (*n* = 42), moderately poor (*n* = 35), and Poor (*n* = 2).

To further explore this relationship, we included all participants in the analysis and categorized them according to their MED-DQI scores: G1 (good, 1–4), G2 (moderately good, 5–7), G3 (moderately poor, 8–10), and G4 (poor, 11–14). The global inflammatory index, calculated as the average of 45 standardized cytokines, showed an apparent increase from G1 to G4 (Figure 2). This analysis should be considered descriptive and exploratory.

**Figure 3:**
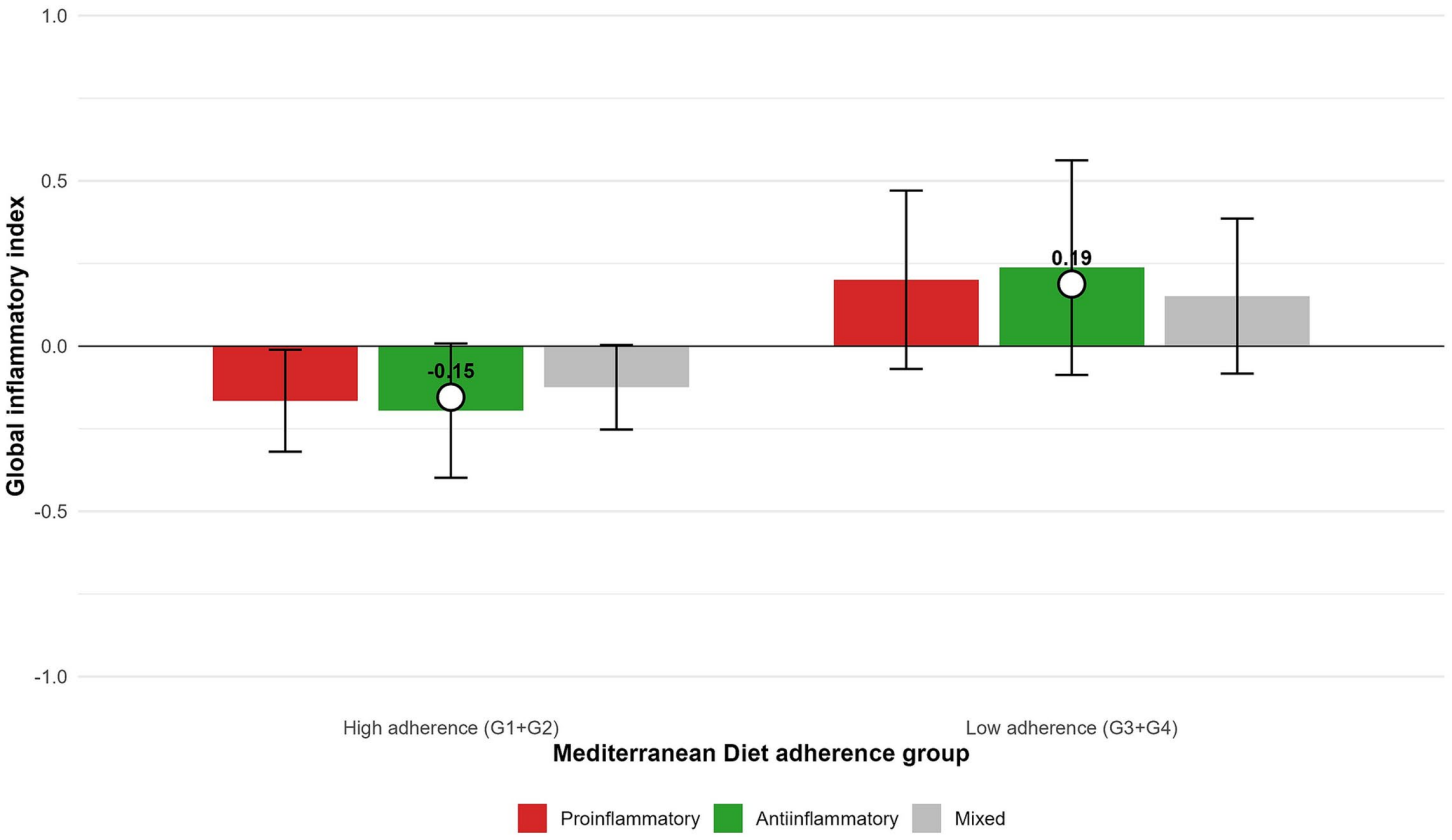
Inflammatory profile and Mediterranean diet adherence [higher (G1 + G2) vs. lower (G3 + G4)]. Participants were classified as higher adherence (G1 + G2) or lower adherence (G3 + G4). Cytokines were grouped as pro-inflammatory, anti-inflammatory, or mixed. White circles represent the mean of Global inflammatory index (±95% CI; *n* = 82).

However, because the number of participants in the extreme categories (G1 and G4) was small, we regrouped individuals into two broader categories for a confirmatory analysis: higher adherence (G1 + G2) and lower adherence (G3 + G4). Those with lower adherence to the Mediterranean diet (G3 + G4) showed a significantly higher global inflammatory index compared with those with higher adherence (Student’s *t* = −2.46; df = 80; *p* = 0.016). The mean difference was −0.34 (95% CI: −0.62 to −0.07). In addition, participants in the lower-adherence group exhibited significantly higher concentrations of several pro-inflammatory, anti-inflammatory, and mixed cytokines, suggesting a pattern consistent with persistent low-grade inflammation (Figure 3).

### 3.4 Association between Mediterranean diet adherence and an elevated inflammatory index: odds ratio analysis

We examined the association between adherence to the Mediterranean diet and inflammatory status using an odds ratio (OR) analysis. To estimate the odds of an elevated inflammatory index, defined as values above the median of global inflammatory index, we conducted an odds ratio (OR) analysis comparing participants with lower (MED-DQI ≥ 8) and higher (MED-DQI ≤ 7) adherence to the Mediterranean diet.

**Figure 4:**
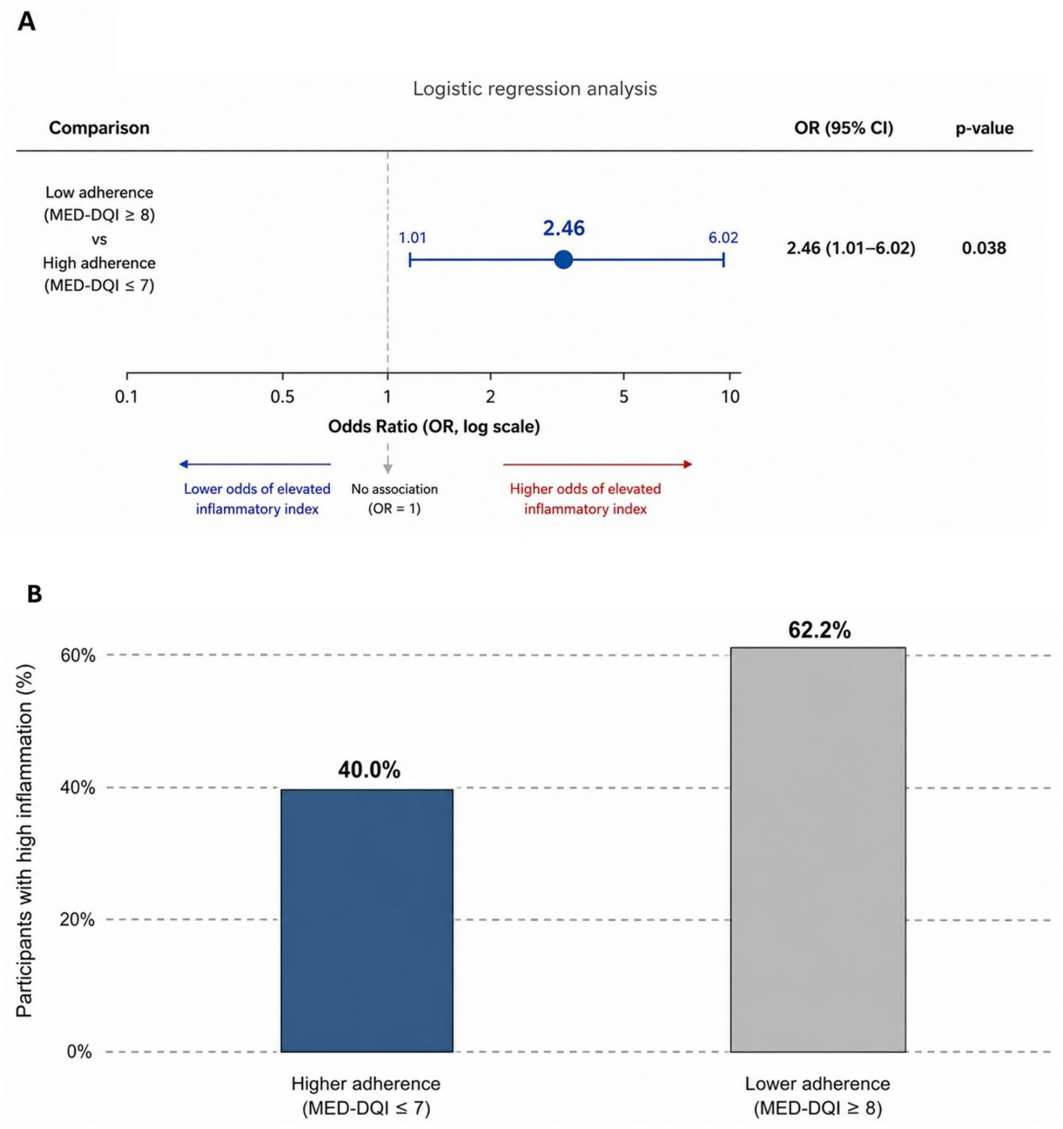
**(A)** Association between Mediterranean diet adherence and the odds of an elevated inflammatory index. Forest plot showing the OR (95% CI) from the logistic regression analysis comparing low adherence (MED-DQI ≥ 8) versus high adherence (MED-DQI ≤ 7). The dashed vertical line indicates no association (OR = 1). **(B)** Elevated inflammatory index according to Mediterranean diet adherence. Percentage of participants with an elevated inflammatory index in the higher adherence (MED-DQI ≤ 7) and lower adherence (MED-DQI ≥ 8) groups.

Participants with low adherence (MED-DQI ≥ 8) had higher odds of presenting a high inflammatory profile (Q3–Q4 mean ln *Z*-score of cytokines) compared with those with better adherence (MED-DQI ≤ 7) (OR = 2.46; 95% CI: 1.01–6.02; *p* = 0.038) (Figure 4A). This association was consistent with the descriptive distribution of participants across groups, with 62.2% of participants in the low-adherence group and 40.0% in the high-adherence group classified as having an elevated inflammatory index (Figure 4B). Adjustment for age, BMI, alcohol intake, total energy intake, and recreational drug use did not modify the effect estimate, suggesting that these variables did not act as confounding factors.

### 3.5 Linear regressions between MED-DQI, the global inflammatory index, and cytokine *Z*-scores (ln-transformed)

We conducted multiple linear regression analyses to evaluate the association between MED-DQI scores and plasma cytokine concentrations.

First, a regression model using the composite global inflammatory index as the dependent variable showed that higher MED-DQI scores, indicating poorer adherence to the Mediterranean dietary pattern, were associated with higher values of the global inflammatory index (*β* = 0.115, *p* = 0.006, *R*^2^ = 0.091). Adjustment for age, CAFI score, BMI, alcohol intake, total energy intake, and drug use did not materially modify the observed association, suggesting that these variables did not act as relevant confounding factors.

**Figure 5:**
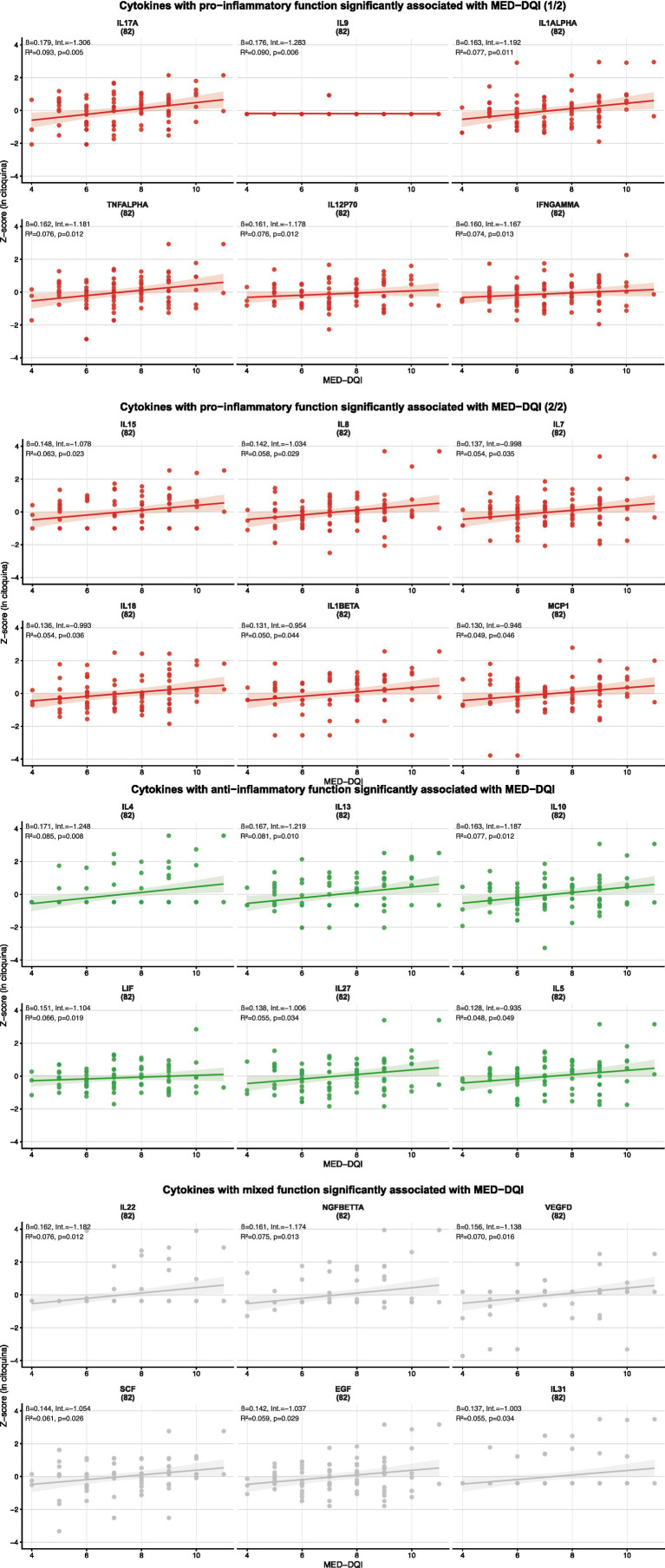
Associations between MED-DQI and cytokine expression (*Z*-scores of ln-transformed concentrations). Each panel shows a linear regression between MED-DQI and cytokine levels. Cytokines are classified as pro-inflammatory, anti-inflammatory, or mixed. Shaded areas represent 95% confidence intervals.

Individual cytokine analyses showed consistent results. Of the 45 cytokines analyzed, 24 were significantly associated with MED-DQI (*p* < 0.05 and qFDR < 0.10) (Figure 5). Across the regression models, higher MED-DQI scores were consistently associated with higher concentrations of inflammatory cytokines, supporting cytokine profile compatible with greater systemic immune activation in participants with lower adherence to the Mediterranean dietary pattern.

After adjustment for age, CAFI score, BMI, alcohol intake, total energy intake and drug use, most associations remained statistically significant. For SCF, EGF, IL-1β, IL-8 and MCP-1, statistical significance decreased slightly, although *Δβ* values remained below 8%, suggesting minimal confounding. IL-5, IL-7, IL-27, IL-31, IL-1*α*, and TNF-α showed *Δβ* values ranging from 10 to 17%, suggesting a modest confounding effect.

### 3.6 Estimated change in cytokine concentrations per one-point increase in MED-DQI

**Figure 6:**
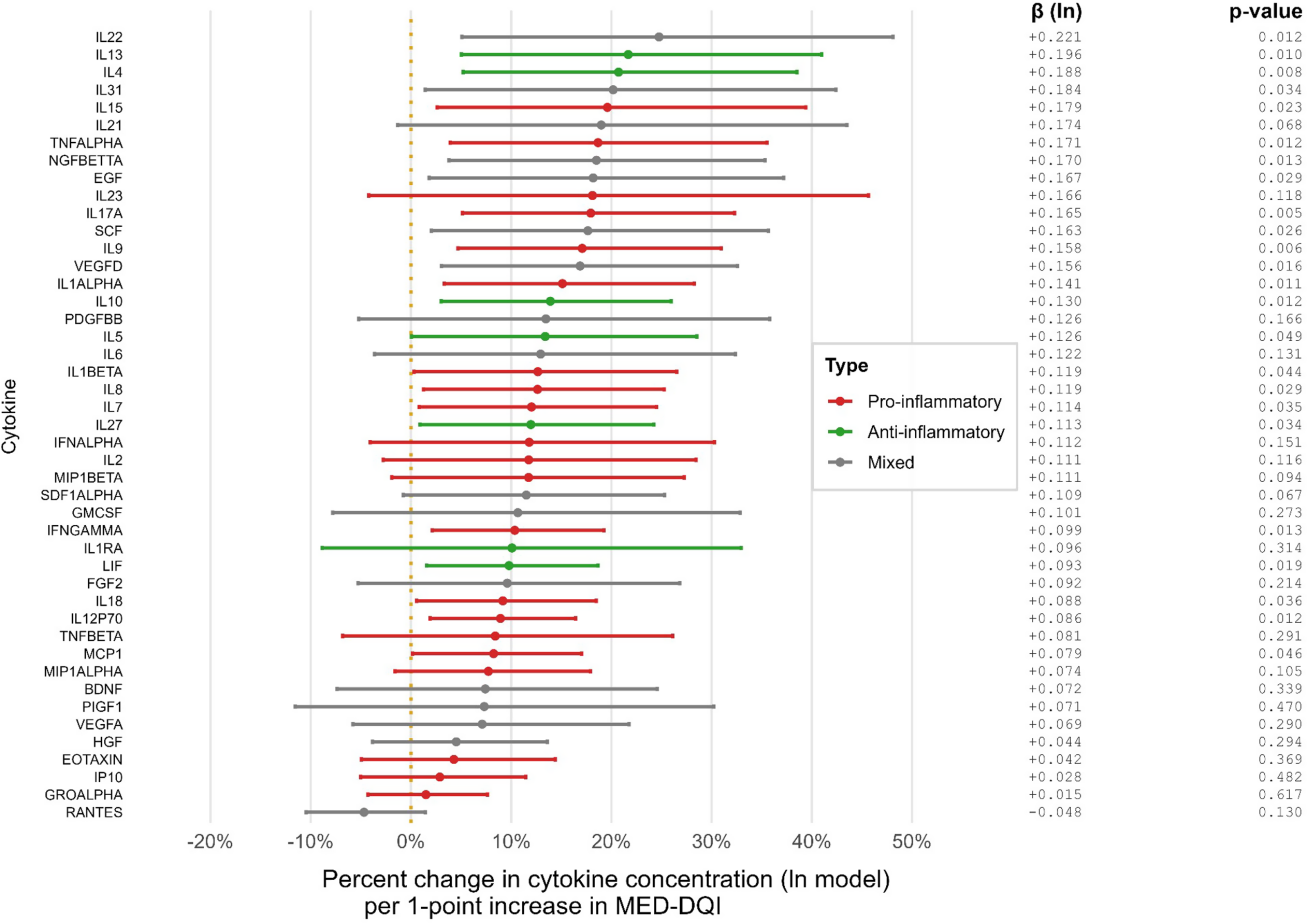
Effect of the MED-DQI on ln-transformed cytokine concentrations. Points represent *β* coefficients (ln scale) with 95% confidence intervals, indicating percent change in cytokine levels per 1-point increase in MED-DQI. Colors indicate cytokine class (pro-inflammatory, anti-inflammatory, mixed).

To provide clinically interpretable effect estimates, we fitted linear regression models using ln-transformed cytokine concentrations (Figure 6). Each point represents the *β* coefficient for a one-point increase in MED-DQI, with horizontal bars indicating 95% confidence intervals (CIs). All *β* coefficients were positive, indicating that poorer diet quality (higher MED-DQI scores) was consistently associated with higher cytokine concentrations.

To enhance interpretability, we converted ln-scale regression coefficients into proportional percentage changes using the formula %*Δ* = (e^(*β*·k) − 1) × 100, where *β* represents the regression coefficient and k the number of MED-DQI points of change.

Each one-point increase in MED-DQI corresponded to a proportional rise in cytokine concentrations, depending on the magnitude of *β*. For example, IL-17A increased by approximately 18% per one-point increase in MED-DQI (*β* = 0.165), with larger MED-DQI differences (*k* = 2 or 3) corresponded to progressively greater increases (approximately 39 and 64%, respectively).

## 4 Discussion

We found that adherence to the Mediterranean diet was associated with a lower global inflammatory index in both PWH and PrEP users. Individuals with lower adherence exhibited a higher global inflammatory index and elevated concentrations across multiple cytokines, consistent with a cytokine profile compatible with greater systemic immune activation.

Notably, no significant differences emerged in either the global inflammatory index or the MED-DQI score between PWH and PrEP users, suggesting that the observed inflammatory patterns were more closely associated with diet quality than with group membership. The observed differences in inflammatory status between participants with higher and lower adherence to the Mediterranean diet are consistent with this interpretation.

Participants with lower adherence consistently exhibited elevations across pro-inflammatory, anti-inflammatory, and regulatory cytokines. This pattern suggests a broadly heightened immune activation state rather than a selective increase in pro-inflammatory signaling. Therefore, the global inflammatory index should be interpreted as a summary measure of the overall cytokine profile, which may reflect differences in systemic immune activation, rather than as a purely pro-inflammatory score. The concurrent increase in anti-inflammatory cytokines may reflect compensatory immunoregulatory responses activated in the context of persistent immune activation. In contrast, participants with higher adherence showed uniformly reduced cytokine concentrations, as reflected by negative mean *Z*-scores across all measured cytokines.

This aligns with prior evidence. A systematic review and meta-analysis of 22 randomized controlled trials demonstrated that Mediterranean-style dietary patterns produced the most consistent reductions in IL-6, IL-1β, and CRP compared with vegetarian, semi-vegetarian, or vegan dietary patterns (27).

Our findings are consistent with previous research reporting associations between Mediterranean dietary patterns and lower inflammatory burden across various populations and may indicate similar relationships in PWH and PrEP users.

In both cases, inflammation has important clinical implications, as persistent immune activation and inflammatory imbalance are associated with an increased risk of metabolic, cardiovascular, and neurocognitive comorbidities (28–30). The association between MED-DQI and IL-17A is consistent with the proposed role of Th17 pathways at the interface between diet, gut microbiota, and systemic inflammation, as suggested by previous studies. IL-17A, the hallmark Th17 cytokine, is essential for mucosal defense but contributes to endothelial and metabolic inflammation when chronically elevated (31). These mechanisms are especially relevant in PWH and PrEP users, where mucosal immune disturbances and gut dysbiosis are frequently observed. In PWH, early depletion and dysfunction of Th17 cells, together with structural disruption of the intestinal barrier, promote microbial translocation and sustain chronic immune activation even under suppressive ART (32, 33). Comparable alterations may also occur in PrEP users, although the underlying mechanisms remain less well characterized and could be influenced by lifestyle factors affecting mucosal health. Within this context, it is plausible that greater adherence to a Mediterranean dietary pattern may be associated with improved Th17/Treg balance, enhanced epithelial integrity, and reduced translocation-driven inflammation, as suggested by previous studies (32–35). However, these mechanisms were not directly assessed in the present study and should therefore be regarded as plausible biological hypotheses rather than conclusions derived from our data.

As such, they may help explain the differences observed in our global inflammatory index between participants with higher and lower adherence to the Mediterranean diet.

Experimental evidence shows that Western-type, high-fat diets promote Th17 skewing and IL-17A production, whereas fiber- and SCFA-rich dietary patterns suppress Th17 activity and favor regulatory responses (36–39). The ~18% rise in IL-17A per one-point worsening in MED-DQI is consistent with previous studies linking dietary patterns to Th17-related immune responses. Given the role of IL-17A in chronic inflammation, this association may be relevant to long-term cardiometabolic health in PWH and PrEP users (40–42).

Our findings indicate that even modest differences in adherence to the Mediterranean diet are associated with measurable variation in systemic inflammatory markers in both people with HIV and PrEP users, suggesting an association between diet quality and chronic immune activation. Consistent patterns across individual cytokines and a global inflammatory index suggest that dietary patterns may represent a modifiable factor contributing to long-term immunometabolic regulation in these populations.

Although the cross-sectional design precludes causal inference, and the relatively modest sample size together with the use of self-reported 3-day dietary data may limit generalizability, several additional limitations should be acknowledged. The composite global inflammatory index was designed as an integrated measure of the overall cytokine profile and does not account for the functional heterogeneity or differential biological weighting of individual cytokines. The index should therefore be interpreted as an exploratory summary measure of the overall cytokine profile rather than as a specific measure of inflammatory burden or activity of individual immune pathways. In addition, information regarding smoking habits, socioeconomic factors, and concomitant medications was not systematically available for all participants and therefore could not be included in the adjusted analyses. Nevertheless, the consistency of the observed associations suggests that diet quality may represent a potentially modifiable factor associated with systemic inflammatory profiles in these populations. Future longitudinal and interventional studies integrating comprehensive dietary assessment with microbiome, metabolomic, and mucosal immune profiling will help determine whether improvements in diet quality can modify inflammatory trajectories and reduce chronic disease risk.

## Data Availability

The original contributions presented in the study are included in the article/Supplementary material, further inquiries can be directed to the corresponding authors.
