# Supplementary material for: Mediterranean diet adherence and systemic inflammation in people with HIV and PrEP users
Source: Front Nutr. 2026 Jul 16;13:1860061. doi: 10.3389/fnut.2026.1860061 (PMC13422545; doi:10.3389/fnut.2026.1860061)
Supplement: Supplementary file 1 [file Table_1.pdf]

Supplementary Table 1. Descriptive Statistics and MED-DQI Associations for 45 Cytokines

Cytokine	Detectable n	Mean	Std. Deviation	Coef. Pearson	p value	qFDR	β (95% CI)
**IL17A**	81	7.33	6.96	0.305	0.0054	0.0546	0.179 (0.054, 0.303)
**IL9**	5	4.56	25.33	0.299	0.0063	0.0546	0.176 (0.051, 0.300)
**IL4**	18	4.25	14.28	0.291	0.008	0.0546	0.171 (0.046, 0.296)
**IL13**	50	4.33	8.28	0.284	0.0096	0.0546	0.167 (0.042, 0.292)
**IL1ALPHA**	82	0.43	0.67	0.278	0.0114	0.0546	0.163 (0.038, 0.288)
**IL10**	82	2.57	3.35	0.277	0.0118	0.0546	0.163 (0.037, 0.288)
**TNFALPHA**	80	4.20	7.83	0.276	0.0122	0.0546	0.162 (0.036, 0.287)
**IL22**	13	12.77	55.12	0.276	0.0121	0.0546	0.162 (0.036, 0.287)
**IL12P70**	82	1.23	1.40	0.275	0.0124	0.0546	0.161 (0.036, 0.287)
**NGFBETTA**	25	4.19	16.33	0.274	0.0127	0.0546	0.161 (0.035, 0.286)
**IFNGAMMA**	82	9.47	13.33	0.272	0.0133	0.0546	0.160 (0.034, 0.285)
**VEGFD**	25	0.55	1.78	0.265	0.0159	0.0598	0.156 (0.030, 0.282)
**LIF**	82	3.03	4.66	0.258	0.0194	0.0672	0.151 (0.025, 0.277)
**IL15**	49	7.21	13.42	0.252	0.0226	0.0726	0.148 (0.021, 0.274)
**SCF**	64	1.38	3.03	0.246	0.0259	0.0765	0.144 (0.018, 0.271)
**EGF**	82	54.02	142.04	0.242	0.0286	0.0765	0.142 (0.015, 0.269)
**IL8**	81	1.24	2.55	0.241	0.0289	0.0765	0.142 (0.015, 0.268)
**IL27**	82	45.22	72.57	0.235	0.0337	0.0775	0.138 (0.011, 0.265)
**IL31**	13	9.41	34.91	0.234	0.0342	0.0775	0.137 (0.010, 0.264)
**IL7**	82	0.87	1.43	0.233	0.0353	0.0775	0.137 (0.010, 0.264)
**IL18**	82	28.29	23.15	0.232	0.0362	0.0775	0.136 (0.009, 0.263)
**IL1BETA**	53	1.79	2.66	0.223	0.0443	0.0904	0.131 (0.003, 0.258)
**MCP1**	80	11.96	7.93	0.221	0.0462	0.0904	0.130 (0.002, 0.257)
**IL5**	78	8.06	15.47	0.218	0.049	0.0919	0.128 (0.001, 0.255)
**SDF1ALPHA**	81	179.93	196.39	0.204	0.0665	0.1184	0.119 (-0.008, 0.247)
**IL21**	53	11.39	26.60	0.202	0.0684	0.1184	0.119 (-0.009, 0.246)
**MIP1BETA**	72	11.92	12.40	0.186	0.0937	0.1299	0.109 (-0.019, 0.238)
**MIP1ALPHA**	81	0.45	0.37	0.18	0.1051	0.1299	0.106 (-0.023, 0.234)
**IL2**	69	9.43	17.20	0.175	0.116	0.1299	0.103 (-0.026, 0.231)
**IL23**	42	18.74	48.02	0.174	0.118	0.1299	0.102 (-0.026, 0.231)
**IL6**	70	16.31	38.23	0.168	0.131	0.1299	0.099 (-0.030, 0.227)
**IFNALPHA**	71	0.42	0.92	0.16	0.1507	0.1299	0.094 (-0.035, 0.223)
**PDGFBB**	82	147.67	213.50	0.155	0.1657	0.1299	0.091 (-0.038, 0.220)
**fgf2**	50	3.08	3.89	0.139	0.2141	0.1299	0.081 (-0.048, 0.211)
**GMCSF**	64	17.21	31.53	0.123	0.2728	0.1299	0.072 (-0.058, 0.201)
**VEGFA**	82	45.33	64.07	0.118	0.2901	0.1299	0.069 (-0.060, 0.199)
**TNFBETA**	35	3.22	6.31	0.118	0.2911	0.1299	0.069 (-0.060, 0.199)
**hgf**	82	25.47	22.42	0.117	0.2944	0.1299	0.069 (-0.061, 0.198)
**IL1RA**	78	300.30	350.51	0.113	0.3141	0.1299	0.066 (-0.064, 0.196)
**BDNF**	82	13.76	30.50	0.107	0.339	0.1299	0.063 (-0.067, 0.192)
**EOTAXIN**	78	6.16	7.61	0.1	0.3692	0.1299	0.059 (-0.071, 0.189)
**PIGF1**	77	2.42	3.48	0.081	0.47	0.1299	0.047 (-0.083, 0.178)
**IP10**	80	5.35	3.98	0.079	0.482	0.1299	0.046 (-0.084, 0.176)
**GROALPHA**	77	2.01	1.29	0.056	0.6168	0.1299	0.033 (-0.097, 0.163)
**RANTES**	82	15.34	7.48	-0.169	0.1299	0.1299	-0.099 (-0.228, 0.030)

For each cytokine, the table reports detectable n, mean concentration, SD, Pearson's r, p-value, qFDR, and β (95% CI). Analyses were performed using ln-transformed cytokine concentrations.
